# Our New York Letter

**Published:** 1885-02

**Authors:** Hunter P. Cooper

**Affiliations:** Presbyterian Hospital, New York


### Correspondence.

### OUR NEW YORK LETTER.

ASIATIC CHOLERA.—NEW YORK ORTHOPEDIC SOCIETY.—HYDROCHLORATE OF COCAINE.—CHARGES AGINST DR. FORDYCE BARKER, PRESIDENT OF THE NEW YORK ACADEMY OF MEDICINE.

PRESBYTERIAN HOSPITAL, NEW YORK.

*Editors Atlanta Medical and Surgical Journal:*

One of the most absorbing topics among New York physicians at present, is Asiatic cholera.

The County Medical Association has recently devoted an entire meeting to the discussion of this subject, the paper of the evening being by Prof. Janeway. It is the almost unanimous opinion that we will have a visitation from this scourge next spring and summer, and hence the deep interest that pervades the medical community.

The city government is practically as indifferent on the subject as it could well be. It is true that the Health Board has an appropriation from the common council of $50,000, "to be used in case of an epidemic," but the only true way to fight the cholera is to put the city in the best hygienic condition before it arrives. Scrupulous sanitation is greatly needed in advance. The city at present is in a most deplorable condition. Of course Broadway, Fifth Ave., Madison Ave., and all the streets along which the better classes live, are clean enough. But it is in the tenement-house districts, where the poor are huddled together like sheep, that epidemics rage most violently

My duties as a hospital physician call me almost daily to the sick in tenement houses, and I often wonder how it is possible that human beings can live in such squalor as surrounds the poor of

New York city. In these districts the streets are often reeking with the foul odors from heaps of decaying garbage that the city authorities neglect to remove. The interior of the houses is even still worse; the moment you enter, your fingers involuntarily compress your nostrils. The rooms are small, badly ventilated, badly lighted. and teeming with human and *insect* life. There is a water closet on each floor, and from each a horrible stench emanates; or else there is a large privy vault in the yard, which is used by all the tenants. The system of drainage, by which the refuse is carried from the house to the sewer. is usually crude and imperfect. Should the cholera visit us, these will be the festering plague spots, and the victims will be counted by the thousands.

It is to be hoped that the people of Atlanta will place their fair city in the best sanitary condition against a possible cholera epidemic.

Dr. Janeway, in his paper on cholera before the County Medical Association, Dec. 15th, discussed the nature, etiology, prevention and treatment of the disease. He believes it to be due to a peculiar and specific poison, which is either inherent in, or generated, by the "comma bacilli" of Koch. He has great faith in the investigations of Koch. In the light of Koch's very recent experiments, by which the ingestion of pure cultures of comma bacilli caused cholera, Dr. Janeway believes the disease to be directly communicable from one individual to another. The numerous cases in which persons have swallowed cholera poison without contracting the disease, he explains on the ground that they were insusceptible at that particular time. The same thing, he says, is seen in some persons who are repeatedly exposed to small-pox and typhus fever, and yet never have either disease. He believes the comma bacilli to be the cause of cholera. As preventive measures he advocates strict quarantine, and thorough cleanliness of the city. Every privy vault that is unconnected with the sewer, should be cleaned out, disinfected, and if possible, closed up. If the disease has appeared in a house, after the recovery or death of the patient, that house should be thoroughly fumigated with sulphur, and then ventilated. All cholera dejections should at once be disinfected. He knows of no better treatment for the patient than that advised by Dr. Flint.

The President, Dr. Detmold, then made a few remarks. He was inclined to doubt the potent influence of the comma bacillus. Like David, "he had been young, but now he is old," and in his day and generation he had seen so many plausible theories appear and vanish, that now he was very slow to give any discovery credence until it had been thoroughly established by long-continued and unbiased investigation.

Dr. Austin Flint, Sr., was then called on, and gave his experience in the epidemic of 1849. He at one time believed the disease non-communicable, but at present he holds the contrary opinion. He strongly urged the immediate treatment of the diarrhœa by which cholera is so often preceded, for by this plan many cases are aborted at the outset. For this purpose he advised the use of opium and astringents.

He was followed by Dr. S. S Purple, ex-President of the Academy of Medicine, who described the outbreak and progress of the epidemic of 1866.

An Orthopedic Society has been recently formed here by the leading orthopedic surgeons. The following are the charter members: Drs. Sayre, Gibney, Schaffer, Yale, Judson, Bull and Wyeth. It is, I believe, the only one in existence in this country, and its transactions will doubtless stimulate the interest in this branch of surgery among practitioners generally.

Hydrochlorate of cocaine still occupies the high place to which it was assigned a few months ago. Every journal contains additional proof of its great value. Several cases of its use have come under my personal observation. In one of these there was corneal ulceration with great pain and photophobia. Its effects were magical; both symptoms disappeared entirely, to the great relief of the patient.

THE CHARGES AGAINST DR. FORDYCE BARKER.

New York physicians feel most acutely the insult recently offered one of the most beloved and famous of their number. I refer to the charges brought against Dr. Fordyce Barker, President of the Academy of Medicine. As the accounts in the journals are very meagre I will tell the whole story:

A few weeks ago Dr. Barker was accused of having registered himself in the Academy as a graduate of the *Ecole de Medicine* of Paris, when he had never received a degree from this institution. The accusation was signed by about six members of the Academy, led by that restless and revengeful spirit, Austin Flint, Jr. The charges were referred to the committee on Ethics. At the last meeting of the Academy the committee reported that after thorough investigation they found the accusation to be utterly false. Their report was unanimously adopted. The facts brought out by the research of the committee are as follows: In 1845 Dr. Barker was a candidate for a degree at the *Ecole de Medicine* in Paris. The illness of his wife suddenly called him home, before he had finished his final examinations or presented a thesis, so that he left Paris without receiving a degree. In 1861 he visited Paris again, and was dining with the famous Trousseau and the Minister of Public Instruction, when the former referred to Dr. Barker as a Paris graduate. Dr. Barker at once disclaimed the honor and narrated the circumstances which compelled him to leave Paris in 1845 without a diploma. Trousseau was greatly surprised, for he remembered having seen the diploma with Dr Barker's name on it and thought it had been sent to him after his return home, then turning to the Minister of Public Instruction he asked him to have the archives of his department searched for the missing diploma. This was done, and in due time Dr Barker received the document, and brought it back to America with him The night of his return home a number of his old friends called on him and all of them saw the diploma. It was dated 1845, and signed by all the members of the faculty of the *Ecole de Medicine.*

In 1876 Dr. Barker moved from his old home in Union Place to his present residence on Madison Avenue. During the transfer of his furniture, books, etc, this diploma and another which he prized very highly were lost.

Dr. Flint, Jr., wrote to Paris, to the Registrar of the College, and asked if Dr. Barker's name could be found on the roll of graduates. The gentleman answered that "the name of Fordyce Barker was not to be found on *the list of those who wrote successful theses for graduation, but it was possible for Dr. Barker to have graduated, although his name did not appear on this list.*"

The Faculty saw fit to issue him a diploma in spite of his having never presented a thesis, hence his name would not appear on the list referred to by the Registrar.

All the friends who welcomed Dr. Barker on the first night of his return home in 1861 are dead, except two. Fortunately these two are men of such irreproachable character that their testimony convinced the Committee of the falseness of the accusation against Dr. Barker. They are Professor Doremus, of Bellevue College, and Dr. Davis, an old and highly esteemed physician of this city. Both of them had seen the diploma and read it through in 1861. Such evidence as this, of course, utterly disproved the charge.

The indignation against Dr. Barker's accusers was so great that a motion was made to expel them from the Academy, but at the earnest request of Dr. Barker himself this motion was not voted on.

Dr. Flint's motive was undoubtedly revenge. Last year, as leader of the "old code" forces, he was defeated in the Academy. Dr. Barker is very strongly in favor of the "new code," and it is believed that Dr. Flint bore him malice, because he was so prominent in the fight last year. This accusation brought against Dr. Barker is not believed by anyone to be the work of the old code party, but simply of Dr. Austin Flint, Jr.

The facts contained in my letter were kindly furnished to me by one of the Committee on Ethics. Very truly,

HUNTER P. COOPER, M. D.

